# Supplementary material for: SARS-CoV-2 lineage B.6 was the major contributor to early pandemic transmission in Malaysia
Source: PLoS Negl Trop Dis. 2020 Nov 30;14(11):e0008744. doi: 10.1371/journal.pntd.0008744 (PMC7728384; doi:10.1371/journal.pntd.0008744)
Supplement: S1 Table — The first 58 sequences were generated in this study. Sequences 77–80 were published by our centre [9]. (DOCX) [file pntd.0008744.s001.docx]

**S1 Table. List of 115 SARS-CoV-2 genomes derived from Malaysian samples and available in GISAID which were used in this study.** The first 58 sequences were generated in this study.

| **No** | **Virus name** | **GISAID accession ID** | **Collection date** | **Probable source** | **Pangolin lineage** | **Clade**  **(GISAID)** | **Clade (Nextstrain)** | **No. raw reads** | **No. of mapped reads** | **Percent covered (%)** | **Average depth** |
| --- | --- | --- | --- | --- | --- | --- | --- | --- | --- | --- | --- |
| 1 | hCoV-19/Malaysia/6359/2020 | EPI_ISL_501220 | 25-Feb-20 | Travel (Japan) | B | L | 19A | 42547 | 42503 | 99.78 | 402.62 |
| 2 | hCoV-19/Malaysia/0956/2020 | EPI_ISL_501181 | 04-Mar-20 | Local transmission | B | L | 19A | 71264 | 71003 | 99.98 | 670.38 |
| 3 | hCoV-19/Malaysia/7618/2020 | EPI_ISL_501221 | 17-Mar-20 | Travel (Indonesia) | B | L | 19A | 33705 | 32785 | 99.2 | 311.47 |
| 4 | hCoV-19/Malaysia/8816/2020 | EPI_ISL_501224 | 18-Mar-20 | Local transmission | B.6 | O | 19A | 44340 | 44086 | 99.72 | 417.48 |
| 5 | hCoV-19/Malaysia/8454/2020 | EPI_ISL_501223 | 18-Mar-20 | Local transmission | B | L | 19A | 45861 | 45266 | 99.7 | 429.72 |
| 6 | hCoV-19/Malaysia/9593/2020 | EPI_ISL_501226 | 19-Mar-20 | Travel (United Kingdom) | B.3 | L | 19A | 40280 | 39928 | 99.51 | 377.47 |
| 7 | hCoV-19/Malaysia/9136/2020 | EPI_ISL_501225 | 19-Mar-20 | Local transmission | B.6 | O | 19A | 35632 | 35534 | 99.78 | 335.28 |
| 8 | hCoV-19/Malaysia/9857/2020 | EPI_ISL_501227 | 21-Mar-20 | Local transmission | B.6 | O | 19A | 51341 | 51177 | 99.78 | 483.38 |
| 9 | hCoV-19/Malaysia/0121/2020 | EPI_ISL_501176 | 21-Mar-20 | Travel (Spain) | B.1.1 | GR | 20B | 433726 | 432686 | 99.91 | 4248.77 |
| 10 | hCoV-19/Malaysia/9886/2020 | EPI_ISL_501228 | 21-Mar-20 | Local transmission | B.6 | O | 19A | 32288 | 32161 | 99.85 | 304.27 |
| 11 | hCoV-19/Malaysia/0309/2020 | EPI_ISL_501177 | 22-Mar-20 | Travel (United Kingdom) | B.1.1 | GR | 20B | 60459 | 60010 | 98.46 | 569.72 |
| 12 | hCoV-19/Malaysia/0784/2020 | EPI_ISL_501179 | 23-Mar-20 | Local transmission | B | L | 19A | 37518 | 37223 | 99.76 | 352.85 |
| 13 | hCoV-19/Malaysia/0478/2020 | EPI_ISL_501178 | 23-Mar-20 | Local transmission | B.6 | O | 19A | 31726 | 31651 | 99.78 | 297.92 |
| 14 | hCoV-19/Malaysia/0931/2020 | EPI_ISL_501180 | 23-Mar-20 | Local transmission (healthcare-associated outbreak) | B.6 | O | 19A | 36522 | 36379 | 99.76 | 345.25 |
| 15 | hCoV-19/Malaysia/1121/2020 | EPI_ISL_501182 | 24-Mar-20 | Travel (Kenya) | B.6 | O | 19A | 34926 | 34814 | 99.78 | 328.77 |
| 16 | hCoV-19/Malaysia/1204/2020 | EPI_ISL_501183 | 24-Mar-20 | Travel (Singapore) | B.6 | O | 19A | 51950 | 51849 | 99.89 | 489.34 |
| 17 | hCoV-19/Malaysia/1713/2020 | EPI_ISL_501186 | 25-Mar-20 | Local transmission (healthcare-associated outbreak) | B.6 | O | 19A | 42713 | 42636 | 99.82 | 400.8 |
| 18 | hCoV-19/Malaysia/1798/2020 | EPI_ISL_501187 | 25-Mar-20 | Local transmission | B | L | 19A | 49326 | 49116 | 99.86 | 463.23 |
| 19 | hCoV-19/Malaysia/1497/2020 | EPI_ISL_501185 | 25-Mar-20 | Travel (United Kingdom) | B.1.1 | GR | 20B | 39765 | 39607 | 99.78 | 375.01 |
| 20 | hCoV-19/Malaysia/1399/2020 | EPI_ISL_501184 | 25-Mar-20 | Religious mass gathering | B.6 | O | 19A | 64472 | 64055 | 99.78 | 608.39 |
| 21 | hCoV-19/Malaysia/2063/2020 | EPI_ISL_501188 | 26-Mar-20 | Religious mass gathering | B.6 | O | 19A | 40839 | 40663 | 99.76 | 382.8 |
| 22 | hCoV-19/Malaysia/2065/2020 | EPI_ISL_501189 | 26-Mar-20 | Local transmission (healthcare-associated outbreak) | B.6 | O | 19A | 39250 | 39151 | 99.78 | 366.14 |
| 23 | hCoV-19/Malaysia/2079/2020 | EPI_ISL_501190 | 26-Mar-20 | Local transmission (healthcare-associated outbreak) | B.6 | O | 19A | 43363 | 43272 | 99.89 | 407.68 |
| 24 | hCoV-19/Malaysia/2363/2020 | EPI_ISL_501194 | 27-Mar-20 | Local transmission (healthcare-associated outbreak) | B.6 | O | 19A | 47159 | 47049 | 99.78 | 443.53 |
| 25 | hCoV-19/Malaysia/2297/2020 | EPI_ISL_501193 | 27-Mar-20 | Local transmission | B.6 | O | 19A | 59392 | 59221 | 99.9 | 561.4 |
| 26 | hCoV-19/Malaysia/2875/2020 | EPI_ISL_501198 | 28-Mar-20 | Religious mass gathering | B.6 | O | 19A | 52286 | 52182 | 99.86 | 491.49 |
| 27 | hCoV-19/Malaysia/2811/2020 | EPI_ISL_501196 | 28-Mar-20 | Local transmission | B.6 | O | 19A | 45799 | 45679 | 99.89 | 426.84 |
| 28 | hCoV-19/Malaysia/2735/2020 | EPI_ISL_501195 | 28-Mar-20 | Local transmission (healthcare-associated outbreak) | B.6 | O | 19A | 50720 | 50584 | 99.96 | 473.83 |
| 29 | hCoV-19/Malaysia/2813/2020 | EPI_ISL_501197 | 28-Mar-20 | Local transmission (healthcare-associated outbreak) | B.6 | O | 19A | 7908 | 7898 | 99.78 | 74.95 |
| 30 | hCoV-19/Malaysia/3012/2020 | EPI_ISL_501199 | 29-Mar-20 | Local transmission | B.6 | O | 19A | 733738 | 724362 | 99.9 | 7119.37 |
| 31 | hCoV-19/Malaysia/3321/2020 | EPI_ISL_501203 | 30-Mar-20 | Unknown | B.6 | O | 19A | 34659 | 34483 | 99.28 | 327.43 |
| 32 | hCoV-19/Malaysia/3499/2020 | EPI_ISL_501205 | 30-Mar-20 | Local transmission | B.6 | O | 19A | 44170 | 44020 | 99.78 | 417.41 |
| 33 | hCoV-19/Malaysia/3145/2020 | EPI_ISL_501202 | 30-Mar-20 | Local transmission (healthcare-associated outbreak) | B.6 | O | 19A | 44186 | 44148 | 99.78 | 417.71 |
| 34 | hCoV-19/Malaysia/3133/2020 | EPI_ISL_501201 | 30-Mar-20 | Local transmission | B.6 | O | 19A | 53681 | 53523 | 99.78 | 506.21 |
| 35 | hCoV-19/Malaysia/3479/2020 | EPI_ISL_501204 | 30-Mar-20 | Travel (United Kingdom) | B.1.1 | GR | 20B | 48240 | 48085 | 99.89 | 454.37 |
| 36 | hCoV-19/Malaysia/3706/2020 | EPI_ISL_501209 | 31-Mar-20 | Local transmission (healthcare-associated outbreak) | B.6 | O | 19A | 45002 | 44951 | 99.82 | 422.46 |
| 37 | hCoV-19/Malaysia/3611/2020 | EPI_ISL_501207 | 31-Mar-20 | Travel (United Kingdom) | B.1.1 | GR | 20B | 32432 | 32391 | 99.78 | 305.63 |
| 38 | hCoV-19/Malaysia/3703/2020 | EPI_ISL_501208 | 31-Mar-20 | Local transmission (healthcare-associated outbreak) | B.6 | O | 19A | 41330 | 41263 | 99.78 | 387.17 |
| 39 | hCoV-19/Malaysia/3605/2020 | EPI_ISL_501206 | 31-Mar-20 | Travel (United Kingdom) | B.2 | V | 19A | 253283 | 220200 | 99.43 | 2160.33 |
| 40 | hCoV-19/Malaysia/3998/2020 | EPI_ISL_501210 | 01-Apr-20 | Religious mass gathering | B.6 | O | 19A | 45503 | 45390 | 99.78 | 426.58 |
| 41 | hCoV-19/Malaysia/5056/2020 | EPI_ISL_501211 | 02-Apr-20 | Travel (United Kingdom) | B.2 | V | 19A | 42401 | 42330 | 99.79 | 397.25 |
| 42 | hCoV-19/Malaysia/5425/2020 | EPI_ISL_501212 | 04-Apr-20 | Local transmission (healthcare-associated outbreak) | B.6 | O | 19A | 46610 | 46486 | 99.86 | 437.5 |
| 43 | hCoV-19/Malaysia/5906/2020 | EPI_ISL_501215 | 06-Apr-20 | Local transmission (healthcare-associated outbreak) | B.6 | O | 19A | 40657 | 40579 | 99.89 | 380.13 |
| 44 | hCoV-19/Malaysia/5815/2020 | EPI_ISL_501213 | 06-Apr-20 | Local transmission | B.6 | O | 19A | 51342 | 51284 | 99.78 | 482.52 |
| 45 | hCoV-19/Malaysia/5822/2020 | EPI_ISL_501214 | 06-Apr-20 | Local transmission (healthcare-associated outbreak) | B.6 | O | 19A | 38876 | 38793 | 99.83 | 364.42 |
| 46 | hCoV-19/Malaysia/6149/2020 | EPI_ISL_501217 | 06-Apr-20 | Local transmission | B.6 | O | 19A | 42449 | 42388 | 99.78 | 397.96 |
| 47 | hCoV-19/Malaysia/6088/2020 | EPI_ISL_501216 | 06-Apr-20 | Local transmission | B.6 | O | 19A | 215958 | 195737 | 99.26 | 1920.93 |
| 48 | hCoV-19/Malaysia/6216/2020 | EPI_ISL_501218 | 07-Apr-20 | Local transmission | B.6 | O | 19A | 449131 | 444230 | 99.9 | 4367.74 |
| 49 | hCoV-19/Malaysia/6306/2020 | EPI_ISL_501219 | 07-Apr-20 | Local transmission | B.6 | O | 19A | 53512 | 53234 | 99.78 | 504.7 |
| 50 | hCoV-19/Malaysia/7685/2020 | EPI_ISL_506996 | 10-Apr-20 | Local transmission | B.6 | O | 19A | 290798 | 261116 | 99.9 | 2561.73 |
| 51 | hCoV-19/Malaysia/7924/2020 | EPI_ISL_507000 | 10-Apr-20 | Local transmission | B.6 | O | 19A | 242576 | 130589 | 90.42 | 1249.19 |
| 52 | hCoV-19/Malaysia/7970/2020 | EPI_ISL_506997 | 11-Apr-20 | Local transmission | B.6 | O | 19A | 287275 | 137600 | 93.81 | 1294.59 |
| 53 | hCoV-19/Malaysia/8451/2020 | EPI_ISL_501222 | 12-Apr-20 | Travel (Americas) | B.1 | G | 20A | 146142 | 142927 | 99.85 | 1395.57 |
| 54 | hCoV-19/Malaysia/3097/2020 | EPI_ISL_501200 | 04-May-20 | Local transmission | B.6 | O | 19A | 441583 | 439397 | 99.91 | 4300.7 |
| 55 | hCoV-19/Malaysia/2982/2020 | EPI_ISL_506998 | 29-Mar-20 | Local transmission (healthcare-associated outbreak) | B.6 | O | 19A | 61653 | 56569 | 94.3 | 553.5 |
| 56 | hCoV-19/Malaysia/2251/2020 | EPI_ISL_501192 | 27-Mar-20 | Local transmission (healthcare-associated outbreak) | B.6 | O | 19A | 310702 | 308736 | 99.91 | 3012.73 |
| 57 | hCoV-19/Malaysia/5760/2020 | EPI_ISL_506999 | 23-Apr-20 | Local transmission (healthcare-associated outbreak) | B.6 | O | 19A | 147641 | 82018 | 94.24 | 796.88 |
| 58 | hCoV-19/Malaysia/2101/2020 | EPI_ISL_501191 | 26-Mar-20 | Local transmission (healthcare-associated outbreak) | B.6 | O | 19A | 164955 | 162989 | 99.91 | 1582.45 |
| 59 | hCoV-19/Malaysia/IMR_WC119/2020 | EPI_ISL_455790 | 30-Jan-20 | Travel (China) | A | S | 19B | Unknown | Unknown | Unknown | 2202 |
| 60 | hCoV-19/Malaysia/MKAK-CL-2020-6430/2020 | EPI_ISL_416886 | 04-Feb-20 | Travel (China) | A | S | 19B | Unknown | Unknown | Unknown | 133 |
| 61 | hCoV-19/Malaysia/MKAK-CL-2020-5096/2020 | EPI_ISL_416885 | 30-Jan-20 | Travel (China) | A | S | 19B | Unknown | Unknown | Unknown | 79 |
| 62 | hCoV-19/Malaysia/IMR_WC413/2020 | EPI_ISL_455791 | 08-Feb-20 | Unknown | B | L | 19A | Unknown | Unknown | Unknown | 2321 |
| 63 | hCoV-19/Malaysia/IMR_WC458/2020 | EPI_ISL_455792 | 09-Feb-20 | Travel (China) | B | L | 19A | Unknown | Unknown | Unknown | 2062 |
| 64 | hCoV-19/Malaysia/MKAK-CL-2020-5047/2020 | EPI_ISL_416866 | 24-Jan-20 | Travel (China) | B | L | 19A | Unknown | Unknown | Unknown | 160 |
| 65 | hCoV-19/Malaysia/MKAK-CL-2020-7554/2020 | EPI_ISL_416907 | 06-Feb-20 | Travel (China) | B | L | 19A | Unknown | Unknown | Unknown | 116 |
| 66 | hCoV-19/Malaysia/MKAK-CL-2020-5049/2020 | EPI_ISL_416884 | 24-Jan-20 | Travel (China) | B | L | 19A | Unknown | Unknown | Unknown | 2380 |
| 67 | hCoV-19/Malaysia/MKAK-CL-2020-5045/2020 | EPI_ISL_416829 | 24-Jan-20 | Travel (China) | B | L | 19A | Unknown | Unknown | Unknown | 140 |
| 68 | hCoV-19/Malaysia/IMR_WC1114/2020 | EPI_ISL_455793 | 29-Feb-20 | Unknown | B | L | 19A | Unknown | Unknown | Unknown | 1990 |
| 69 | hCoV-19/Malaysia/IMR_WC1177/2020 | EPI_ISL_430439 | 05-Mar-20 | Local transmission | B | L | 19A | Unknown | Unknown | Unknown | Unknown |
| 70 | hCoV-19/Malaysia/IMR_WC1170/2020 | EPI_ISL_430440 | 05-Mar-20 | Local transmission | B | L | 19A | Unknown | Unknown | Unknown | Unknown |
| 71 | hCoV-19/Malaysia/IMR_WC1097/2020 | EPI_ISL_430441 | 29-Feb-20 | Local transmission | B | L | 19A | Unknown | Unknown | Unknown | Unknown |
| 72 | hCoV-19/Malaysia/IMR_WC1098/2020 | EPI_ISL_430442 | 29-Feb-20 | Travel (China) | B | L | 19A | Unknown | Unknown | Unknown | Unknown |
| 73 | hCoV-19/Malaysia/IMR_WC085/2020 | EPI_ISL_430443 | 28-Jan-20 | Travel (China) | B | L | 19A | Unknown | Unknown | Unknown | Unknown |
| 74 | hCoV-19/Malaysia/IMR_WC627/2020 | EPI_ISL_430444 | 12-Feb-20 | Travel (China) | B | L | 19A | Unknown | Unknown | Unknown | Unknown |
| 75 | hCoV-19/Malaysia/IIUM91/2020 | EPI_ISL_455313 | 02-Apr-20 | Unknown | B.1.36 | GH | 20A | Unknown | Unknown | Unknown | Unknown |
| 76 | hCoV-19/Malaysia/IIUM316/2020 | EPI_ISL_455312 | 09-Apr-20 | Unknown | B.6 | O | 19A | Unknown | Unknown | Unknown | Unknown |
| 77 | hCoV-19/Malaysia/189332/2020 | EPI_ISL_417917 | 20-Mar-20 | Local transmission | B.6 | O | 19A | 1,895,160 | 1,821,267 | 99.9 | 5,289 |
| 78 | hCoV-19/Malaysia/188407/2020 | EPI_ISL_417918 | 18-Mar-20 | Case who attended religious mass gathering | B.6 | O | 19A | 2,057,020 | 1,952,563 | 99.9 | 5,696 |
| 79 | hCoV-19/Malaysia/186197/2020 | EPI_ISL_417919 | 14-Mar-20 | Travel (Vietnam) | B | L | 19A | 1,467,222 | 369,427 | 85.05 | 1,087 |
| 80 | hCoV-19/Malaysia/190300/2020 | EPI_ISL_417920 | 22-Mar-20 | Unknown | B.6 | O | 19A | 1,796,760 | 1,647,233 | 99.12 | 4,986 |
| 81 | hCoV-19/Malaysia/IMR_WC2423 | EPI_ISL_459953 | 16-Mar-20 | Unknown | B.6 | O | 19A | Unknown | Unknown | Unknown | Unknown |
| 82 | hCoV-19/Malaysia/IMR_WC2453 | EPI_ISL_459954 | 16-Mar-20 | Unknown | B.6 | O | 19A | Unknown | Unknown | Unknown | Unknown |
| 83 | hCoV-19/Malaysia/IMR_WC2665 | EPI_ISL_459955 | 18-Mar-20 | Unknown | B.6 | O | 19A | Unknown | Unknown | Unknown | Unknown |
| 84 | hCoV-19/Malaysia/IMR_WC9127 | EPI_ISL_459956 | 30-Mar-20 | Unknown | B.6 | O | 19A | Unknown | Unknown | Unknown | Unknown |
| 85 | hCoV-19/Malaysia/IMR_WC10180 | EPI_ISL_459957 | 01-Apr-20 | Unknown | B.6 | O | 19A | Unknown | Unknown | Unknown | Unknown |
| 86 | hCoV-19/Malaysia/IMR-WC9205 | EPI_ISL_489992 | 04-Mar-20 | Unknown | B.6 | O | 19A | Unknown | Unknown | Unknown | Unknown |
| 87 | hCoV-19/Malaysia/IMR-WC9174/2020 | EPI_ISL_489993 | 04-Mar-20 | Unknown | B.6 | O | 19A | Unknown | Unknown | Unknown | Unknown |
| 88 | hCoV-19/Malaysia/IMR-WC9185/2020 | EPI_ISL_489994 | 30-Mar-20 | Unknown | B.6 | O | 19A | Unknown | Unknown | Unknown | Unknown |
| 89 | hCoV-19/Malaysia/IMR-WC10195/2020 | EPI_ISL_490014 | 10-Apr-20 | Unknown | B.6 | O | 19A | Unknown | Unknown | Unknown | Unknown |
| 90 | hCoV-19/Malaysia/IMR-WC12897/2020 | EPI_ISL_490015 | 10-Apr-20 | Unknown | B.1 | G | 20A | Unknown | Unknown | Unknown | Unknown |
| 91 | hCoV-19/Malaysia/IMR-WC12286/2020 | EPI_ISL_490016 | 10-Apr-20 | Unknown | B.6 | O | 19A | Unknown | Unknown | Unknown | Unknown |
| 92 | hCoV-19/Malaysia/IMR-WC13946/2020 | EPI_ISL_490047 | 10-Apr-20 | Unknown | B.6 | O | 19A | Unknown | Unknown | Unknown | Unknown |
| 93 | hCoV-19/Malaysia/IMR-WC14227/2020 | EPI_ISL_490048 | 11-Apr-20 | Unknown | B | L | 19A | Unknown | Unknown | Unknown | Unknown |
| 94 | hCoV-19/Malaysia/IMR-WC55122/2020 | EPI_ISL_490089 | 10-May-20 | Unknown | B.1.1.1 | GR | 20B | Unknown | Unknown | Unknown | Unknown |
| 95 | hCoV-19/Malaysia/IMR-WC80031/2020 | EPI_ISL_490090 | 23-May-20 | Unknown | B.6 | O | 19A | Unknown | Unknown | Unknown | Unknown |
| 96 | hCoV-19/Malaysia/IMR-WC80036/2020 | EPI_ISL_490091 | 23-May-20 | Unknown | B.6 | O | 19A | Unknown | Unknown | Unknown | Unknown |
| 97 | hCoV-19/Malaysia/IMR-WC80066/2020 | EPI_ISL_490092 | 23-May-20 | Unknown | B.6 | O | 19A | Unknown | Unknown | Unknown | Unknown |
| 98 | hCoV-19/Malaysia/IMR-WC80107/2020 | EPI_ISL_490093 | 23-May-20 | Unknown | B.6 | O | 19A | Unknown | Unknown | Unknown | Unknown |
| 99 | hCoV-19/Malaysia/IMR-WC80109/2020 | EPI_ISL_490094 | 23-May-20 | Unknown | B.6 | O | 19A | Unknown | Unknown | Unknown | Unknown |
| 100 | hCoV-19/Malaysia/IMR-WC80558/2020 | EPI_ISL_490095 | 23-May-20 | Unknown | B.6 | O | 19A | Unknown | Unknown | Unknown | Unknown |
| 101 | hCoV-19/Malaysia/IMR-WC81523/2020 | EPI_ISL_490096 | 23-May-20 | Unknown | B.6 | O | 19A | Unknown | Unknown | Unknown | Unknown |
| 102 | hCoV-19/Malaysia/IMR-WC81528/2020 | EPI_ISL_490097 | 23-May-20 | Unknown | B.6 | O | 19A | Unknown | Unknown | Unknown | Unknown |
| 103 | hCoV-19/Malaysia/IMR-WC81849/2020 | EPI_ISL_490098 | 23-May-20 | Unknown | B.6 | O | 19A | Unknown | Unknown | Unknown | Unknown |
| 104 | hCoV-19/Malaysia/IMR-WC99023/2020 | EPI_ISL_490099 | 23-May-20 | Unknown | B.6 | O | 19A | Unknown | Unknown | Unknown | Unknown |
| 105 | hCoV-19/Malaysia/IMR-WC99045/2020 | EPI_ISL_490100 | 23-May-20 | Unknown | B.6 | O | 19A | Unknown | Unknown | Unknown | Unknown |
| 106 | hCoV-19/Malaysia/IMR-WC90685/2020 | EPI_ISL_490101 | 29-May-20 | Unknown | B.1.1.1 | GR | 20B | Unknown | Unknown | Unknown | Unknown |
| 107 | hCoV-19/Malaysia/IMR-WC94436/2020 | EPI_ISL_490102 | 29-May-20 | Unknown | B.1 | G | 20B | Unknown | Unknown | Unknown | Unknown |
| 108 | hCoV-19/Malaysia/IMR-WC94764/2020 | EPI_ISL_490103 | 29-May-20 | Unknown | B.1 | GH | 20A | Unknown | Unknown | Unknown | Unknown |
| 109 | hCoV-19/Malaysia/MGI-G873/2020 | EPI_ISL_528738 | 07-Apr-20 | Unknown | B.1.36 | GH | 20A | Unknown | Unknown | Unknown | Unknown |
| 110 | hCoV-19/Malaysia/MGI-M32/2020 | EPI_ISL_528739 | 02-Apr-20 | Unknown | B.6 | O | 19A | Unknown | Unknown | Unknown | Unknown |
| 111 | hCoV-19/Malaysia/MGI-M64/2020 | EPI_ISL_528740 | 02-Apr-20 | Unknown | B.6 | O | 19A | Unknown | Unknown | Unknown | Unknown |
| 112 | hCoV-19/Malaysia/MGI-M71/2020 | EPI_ISL_528741 | 02-Apr-20 | Unknown | B.6 | O | 19A | Unknown | Unknown | Unknown | Unknown |
| 113 | hCoV-19/Malaysia/MGI-M76/2020 | EPI_ISL_528742 | 02-Apr-20 | Unknown | B.6 | O | 19A | Unknown | Unknown | Unknown | Unknown |
| 114 | hCoV-19/Malaysia/MGI-MAEPS54/2020 | EPI_ISL_528743 | 04-Jun-20 | Unknown | B.6 | O | 19A | Unknown | Unknown | Unknown | Unknown |
| 115 | hCoV-19/Malaysia/MGI-MAEPS67/2020 | EPI_ISL_528744 | 04-Jun-20 | Unknown | B.6 | O | 19A | Unknown | Unknown | Unknown | Unknown |
