# Supplementary material for: SARS-CoV-2 lineage B.6 was the major contributor to early pandemic transmission in Malaysia
Source: PLoS Negl Trop Dis. 2020 Nov 30;14(11):e0008744. doi: 10.1371/journal.pntd.0008744 (PMC7728384; doi:10.1371/journal.pntd.0008744)
Supplement: S2 Table — A difference in viral RNA of one log or 10× in the initial template concentration is equivalent to a Ct difference of 3.3. (DOCX) [file pntd.0008744.s002.docx]

**S2 Table. Comparison of threshold cycle (Ct) values obtained using the Berlin Charité RT-qPCR assay (E gene)^1^ and commercial RT-qPCR assay (nsp3 gene; original and updated primers/probes) using 16 patient samples from this or a previous study^2^ categorised by presence/absence of the nsp3-C6310A substitution.** A difference in viral RNA of one log or 10× in the initial template concentration is equivalent to a Ct difference of 3.3.

| **Sample** | **nsp3-C6310A substitution** | **Ct value** | | | | |
| --- | --- | --- | --- | --- | --- | --- |
|  |  | **[1] Commercial RT-qPCR assay with original primers/probes (nsp3)** | **[2] Commercial RT-qPCR assay with updated primers/probes (nsp3)** | **[3] Charité RT-qPCR assay^1^**  **(E gene)** | **Difference between [1] and [2] (equivalent virus load)** | **Difference between [1] and [3] (equivalent virus load)** |
| 189332 | Yes | 36.2 | 24.71 | 22.81 | 11.49 (>1,000×) | 13.39 (>10,000×) |
| 188407 | Yes | 33.99 | 22.14 | 21.41 | 11.85 (>1,000×) | 12.58 (>1,000×) |
| 2063 | Yes | >40 (negative) | 29.92 | 29.67 | 10.08 (>1,000×) | 10.33 (>1,000×) |
| 2811 | Yes | 31.48 | 16.78 | 15.74 | 14.70 (>10,000×) | 15.74 (>10,000×) |
| 5906 | Yes | 36.46 | 26.65 | 32.76 | 9.81 (>100×) | 3.7 (>10×) |
| 6216 | Yes | >40 (negative) | 33.40 | 34.19 | 6.60 (>100×) | 5.81 (>10×) |
| 186197 | No | 38.50 | 38.80 | 37.40 | 0.30 | 1.10 |
| 190300 | No | 32.03 | 31.61 | 31.00 | 0.42 | 1.03 |
| 5815 | No | 32.10 | Not tested | 30.87 | - | 1.23 |
| 7685 | No | 32.18 | 34.85 | 33.51 | 2.67 | 1.33 |
| 2065 | No | 33.71 | 33.25 | Not tested | 0.46 | - |
| 1497 | No | >40 (negative) | >40 (negative) | Not tested | - | - |
| 3499 | No | 35.65 | 36.26 | Not tested | 0.61 | - |
| 3145 | No | 31.95 | 31.57 | Not tested | 0.38 | - |
| 5056 | No | 33.08 | 33.65 | Not tested | 0.57 | - |
| 7685 | No | 33.51 | 34.85 | Not tested | 1.34 | - |

**References**

1. Corman VM, Landt O, Marco, Kaiser, Molenkamp R, Meijer A, et al. Detection of 2019 novel coronavirus (2019-nCoV) by real-time RT-PCR. Eurosurveillance. 2020;25(3):2000045. doi: 10.2807/1560-7917.ES.2020.25.3.2000045.

2. Chong YM, Sam I-C, Ponnampalavanar S, Omar SFS, Kamarulzaman A, Munusamy V, et al. Complete genome sequences of SARS-CoV-2 strains detected in Malaysia. Microbiology Resource Announcements. 2020;9(20): e00383-20. doi: 10.1128/MRA.00383-20.
