# Supplementary material for: SARS-CoV-2 lineage B.6 was the major contributor to early pandemic transmission in Malaysia
Source: PLoS Negl Trop Dis. 2020 Nov 30;14(11):e0008744. doi: 10.1371/journal.pntd.0008744 (PMC7728384; doi:10.1371/journal.pntd.0008744)
Supplement: Attachment — Submitted filename: Response to reviewer.pdf [file pntd.0008744.s004.pdf]

Decision on your PLOS Neglected Tropical Diseases submission (PNTD-D-20-01503) - [EMID:c3c8287800b6bca8]

We thank the reviewers for their constructive comments. We have taken the opportunity to update the numbers and figures based on GISAID data available up to September 20 (in the initial draft, GISAID data was taken up to June 30). This includes the addition of 7 new Malaysian sequences from other centres, added to the previous 108. The update has not changed any of our main findings or conclusions.

Reviewer #1: It is a SARS-CoV-2 retrospective genomic epidemiological study and the authors appear to have taken necessary steps to elucidate their findings as such.
Some clarification/changes regarding the sample sizes is required.
Thank you. As noted by the reviewers several times below, our manuscript did not always clearly distinguish the numbers of total Malaysian sequences available in GISAID from those generated by our study. As stated in S1 Table, 115 SARS-CoV-2 Malaysian genomes are available in GISAID and 58 of these were generated in this study. We have modified the relevant sentences and made it clearer throughout the manuscript, e.g.: "Malaysian sequences analysed comprised the 58 samples from this study and 57 other Malaysian genome sequences available at GISAID (www.gisaid.org) as of September 20, 2020 [8], including 4 previously published by our centre [9]. Therefore, 62 of the Malaysian sequences were from our centre."

Reviewer #2: The objective of the study is clearly presented, the study design is appropriate, the population has been described. However, the sample size is limited compared to the confirmed cases. There are no ethical concerns. No particular statistics analysis have been used.

Reviewer #3: -Are the objectives of the study clearly articulated with a clear testable hypothesis stated?
-Is the study design appropriate to address the stated objectives?
-Is the population clearly described and appropriate for the hypothesis being tested?
-Is the sample size sufficient to ensure adequate power to address the hypothesis being tested?
-Were correct statistical analysis used to support conclusions?
-Are there concerns about ethical or regulatory requirements being met?
Yes

Results</br></br>
-Does the analysis presented match the analysis plan?</br>
-Are the results clearly and completely presented?</br>
-Are the figures (Tables, Images) of sufficient quality for clarity?</br></br>

Reviewer #1: The results require some clarification/changes. There are no major errors in the analysis.

Reviewer #2: The analysis matches the plan. The results are missing some information and also a new schematic chart would be helpful to better understand where the mutations are located.

A comprehensive schematic chart showing mutations has been added as S3 Table.

Reviewer #3: YES

Conclusions</br></br>
-Are the conclusions supported by the data presented?</br>
-Are the limitations of analysis clearly described?</br>
-Do the authors discuss how these data can be helpful to advance our understanding of the topic under study?</br>
-Is public health relevance addressed?</br></br>

Reviewer #1: The conclusions need some clarification/changes and there is acknowledgement of the limitations of the sequencing studies.

Reviewer #2: No limitations are mentioned. The conclusions reflect the data. However, the authors should highlight the importance of this data in the pandemic, how these data advance the global understanding of SARS-CoV-2 mechanisms of infections, spreading and lethality.

Limitations: We have previously included the sentence: "A caveat is that the number of whole genome sequences reported from developing countries in Asia is relatively low and likely underreports the true incidence of B.6. However, it is notable that this lineage is a very minor contributor in developed countries with greater sequencing efforts (Fig 2C)."

We have also added the sentence: "Detailed, publicly available epidemiological data are also not available for most of the B.6 sequences outside our centre, and would be very useful to further support the involvement of Tablighi Jamaat participants."

We have highlighted the importance of this data in understanding spread during the pandemic:

1. In the discussion, we have written that "initial COVID-19 spread and the predominance of lineage B.6 was temporally and epidemiologically associated with the Tablighi Jamaat religious gathering, and led to established community spread during the main wave in Malaysia. Attendees at this event likely spread strains of the B.6 lineage to other countries in the region, including Southeast Asian countries, India and Australia." We have shown the likely importance of this event in Malaysia in spread of this otherwise rare B.6 lineage regionally.
2. We also noted: "The participants spent several days in large indoor congregations with shared eating and sleeping arrangements, which would have led to extensive transmissions."

We hope these (and other statements) have helped improve understanding of spread. We are unable to comment about lethality as we do not have sufficient cases with sequences and known outcomes.

Reviewer #3: YES

Editorial and Data Presentation Modifications?</br>
Use this section for editorial suggestions as well as relatively minor modifications of existing data that would enhance clarity. If the only modifications needed are minor and/or editorial, you may wish to recommend "Minor Revision" or "Accept".

Reviewer #1: (No Response)

Reviewer #2: A new schematic chart would be helpful to better understand where the mutations are located. With this new chart specific for the mutations, the current trees should be changed.

Thank you for this suggestion. A comprehensive schematic chart showing mutations has been added as S3 Table. We have added 7 new Malaysian sequences into S3 Table and new trees were generated.

Reviewer #3: NONE

Summary and General Comments</br></br>
Use this section to provide overall comments, discuss strengths/weaknesses of the study, novelty, significance, general execution and scholarship. You may also include additional comments for the author, including concerns about dual publication, research ethics, or publication ethics. If requesting major revision, please articulate the new experiments that are needed.

Reviewer #1: The authors describe a SARS-CoV-2 genomic epidemiology study that took place in Malaysia and the finding of a specific viral lineage that is circulating in SEA regions. They also find that a certain mutation leads to reduced sensitivity in commercial assays for the virus.
Thanks to the authors to provide all necessary details to gauge the sequencing and analysis protocols used in the study.
It would be very helpful if the significance of the study to researchers and clinicians/diagnostics can be described in greater detail (Abstract and Introduction).

Sentences were added in Abstract and Introduction, e.g.:

1. "Mass gatherings can be significant causes of local and global spread of COVID-19. Shared genomic surveillance can be used to identify SARS-CoV-2 transmission chains to aid prevention and control, and monitor diagnostic molecular assays."

   2. "In particular, we were interested in determining the possible role of the Tablighi Jamaat gathering in regional spread."

Were the 58 patients from the religious gathering? In the Methodology, explicit details are needed on how many of the 58 patients were from the religious gathering in Malaysia.

And how many of the other 50 Malaysian sequences were from the religious gathering? (Is it 39?)

We perhaps did not make this clear enough. With the update and addition of 7 new Malaysian sequences, there are now 57 other Malaysian sequences not generated by our present study. We have also obtained more epidemiological information for some of our cases and now

report that 7 had links to the Tablighi (previously, we reported 3 with links). We modified these sentences in the methodology:

“The analysed Malaysian sequences comprised the 58 samples from this study and 57 other Malaysian genome sequences available at GISAID (www.gisaid.org) as of September 20, 2020 [8], which include 4 previously published by our centre [9]. Therefore, 62 of the Malaysian sequences were from our centre.”

We have modified this sentence in the results:

“Of the 62 cases from our centre, comprising 58 from this study and 4 from an earlier study [9], 7 had direct links to the Tablighi Jamaat gathering - 4 had attended and 3 were contacts of attendees.”

This epidemiological link between the Tablighi and B.6 lineage is critical to establish, and it was notable that all of the 7 cases with known Tablighi links were infected with B.6 viruses. We later make the point that “most of our B.6 cases (were) without direct or discernible links to the Tablighi Jamaat event”, implying that B.6, an otherwise rare lineage, had become established in the community. If the vast majority of B.6 Malaysian sequences were from the Tablighi rather than community cases with no Tablighi links, we would not have been able to deduce that the B.6 lineage had become established in the wider community.

The publicly available epidemiological data for the remaining 53 Malaysian sequences from other centres do not state if they were associated with the religious gathering, although 13 are clearly stated to be travel-related, while 11 predated the event. We have added this sentence in the discussion as a minor limitation:

“Detailed, publicly available epidemiological data are also not available for most of the B.6 sequences outside our centre, which would be very useful to further support the involvement of Tablighi Jamaat participants.”

The total number of B.6 sequences is not consistent between the last paragraph of Methodology and 3rd paragraph of Results.
A detailed split of B.6 sequences can be provided as: sequenced at UMMC + sequenced at other centers in Malaysia + sequenced at other global centers.

The B.6 numbers have been corrected for consistency. This sentence has been added to the methods: “A total of 1,497 B.6 sequences, including 75 Malaysian B.6 sequences (43 from our study) were available in GISAID database…”

Regarding the events that took place and the statement, "of other Malaysian sequences in GISAID dating from the Tablighi Jamaat gathering".
In the above statement, the timeline of events are not clear.
Also, in the recently published work of the authors "Complete Genome Sequences of SARS-CoV-2 Strains Detected in Malaysia", Fig. 1A the religious event is dated as 29 Feb - 3 Mar, but in the current study the date is 27 Feb - 1 Mar.
Thus, the authors should describe with detail and certainty about the events that took place and that the sequences were from infections originating at the religious gathering.

Rearranging some final sections of the Methodology and beginning of Results could be helpful.

We apologise for the discrepancies in dates; this reflects different reporting sources. For this paper, we have used the dates (27 Feb to 3 Mar) reported by the Malaysian Ministry of Health, and we have ensured this is consistent throughout the manuscript.

Thank you for the suggestion, we have rearranged the results and modified certain sentences for clarity, with citation of the relevant figures, including:

"The Tablighi Jamaat lasted from February 27 to March 3, 2020, and the first reported B.6 sequences globally were from Malaysia, Philippines and Taiwan on March 4 (Fig 2A). Within a week of the Tablighi Jamaat ending, there was a large increase in reported COVID-19 cases in Malaysia, with the vast majority being associated with this gathering. This was accompanied by a spike of lineage B.6 in Malaysia and other countries (Fig 2A and 2B). From March 3 onwards, lineage B.6 sequences comprised 46/61 (75.4%) of sequences from our centre and 29/39 (74.4%) of sequences submitted by other Malaysian centres, indicating that this finding was not specific to our centre. This increase in cases led to the imposition of a nationwide movement control order on March 18."

We feel the timeline of events is now clearer.

Fig.1: If there are 39 sequences from the religious gathering why are only 3 highlighted? To avoid blocking the dendrogram, moving the "red point" to the right side of the sequences would be better.

We apologise for not making clearer the number of sequences with links to the gathering. In our initial draft, we reported 3 with Tablighi links. As mentioned above, we have since obtained more epidemiological information and now report that 7 had links to the Tablighi. We have modified this sentence in the results:

"Of the 62 cases from our centre, comprising 58 from this study and 4 from an earlier study [9], 7 had direct links to the Tablighi Jamaat gathering - 4 had attended and 3 were contacts of attendees."

These seven sequences are now clearly highlighted in Fig 1 and Fig 3.

Fig. 3A: Only an outline of the histogram could be sufficient. This would also make the lineage markers more clear to see.

Fig 3A from the previous draft is now Fig 2A. We have now added data to show both B.6 and non-B.6 lineages and the Tablighi-associated cases reported per day. We hope the figure is now clearer in showing the rise of lineage B.6 was associated with the rise of cases associated with the mass gathering event.

Fig. 3C: Would be helpful to include legend in the figure for Southeast Asian country bars vs other country bars

Thank you for the suggestion. As the spread involved other countries in the Asian region and not just southeast Asian countries, we have classified the countries into Asia Pacific (which includes SE Asia, India and Australia) and others. The legend has been added in the figure.

Reviewer #2: The manuscript "SARS-CoV-2 lineage B.6 is the major contributor to transmission in Malaysia" describes the predominant spreading of the B.6 lineage in Malaysia after a religious mass gathering in Kuala Lumpur. This event is associated with the main wave of COVID-19 cases in Malaysia. The authors analyzed 58 genomes sequences from COVID-19 patients that attended the religious event in Kuala Lumpur and 50 Malaysian sequences available in the GISAID database. The authors detected 9 different lineages, and

among these, the B.6 became the predominant cause of community transmission in Malaysia after the likely introduction during the religious mass gathering.

The references are appropriate.

Major revisions:

- The authors use sometimes SARS-CoV-2 and COVID-19 as synonymous. They are not: SARS-CoV-2 is the novel coronavirus, while COVID-19 includes all the medical conditions caused by SARS-CoV-2 (as also correctly stated by the authors in the introduction). I would suggest the authors to review the manuscript and use the correct terminology.

Thank you, we have reviewed the manuscript and now feel we have used the two terms appropriately.

- The authors should consider adding a more in-depth analysis of the sequences and their mutations. The authors just mentioned a mutation in ORF3a, one in the spike protein, and few more. What about the other viral genes? For example, the one in the polymerase mentioned by Pachetti et al, J Transl Med 18, 179 (2020)?
- A schematic chart would be helpful to better understand where the mutations are located.
- A few typos need to be corrected by the authors.

A comprehensive schematic chart showing mutations has been added as S3 Table. We focused on the key mutations observed in lineage B.6 (which are relatively few and shown in Fig 3) as this lineage is the major cause of local transmission in Malaysia, and hence the main focus of this paper.

The mutations mentioned by Pachetti et al. mostly occurred in Europe and America but were not observed in our Malaysian sequences. We also analysed key receptor binding mutations (41 different rare variants) mentioned in GISAID and these mutations were not observed in our Malaysian sequences. We have included the sentence in the results:

"None of the 41 rare receptor binding variants described in GISAID – including the two most common, S477N and N439K - were observed in the Malaysian sequences."

We did not feel that discussing key mutations in all lineages was within the scope of this paper. There were 40 Malaysian sequences from 8 non-B.6 lineages, which have not shown sustained detection.

Typos: we have carefully checked the final manuscript and corrected all typos.

Reviewer #3: This is a well written manuscript demonstrating how genetic analysis can inform about transmission dynamics of SARS-CoV-2.
The study shows very nicely how a religious gathering fuelled the spread of the B.6 lineage in Malaysia. The results are clearly presented and my only comment to the results section is Fig. 3A where I would prefer to see the results as rates for instance per 100 000 population and not actual number of cases.
Thank you. We reported the results as actual case numbers rather than incidence rates as case numbers were very small compared to Malaysia's population in 2020 of 32.7 million. The

highest reported number of COVID-19 cases per day was only 277, giving a low rate of 0.85 per 100,000. We also felt that readers would be more accustomed to seeing charts with case numbers than rates, as these are commonly shown in the media. We have modified a sentence in the introduction to give readers an idea of the rates:

“In Malaysia, there have been 10,219 confirmed cases with 130 deaths reported as of September 20, 2020 [2], out of a total population of 32.7 million, giving a cumulative incidence of 31 per 100,000.”

However, if the reviewers and editor would like us to change this scale, we would have no objections to doing so.

In the discussion I wonder if the authors could elaborate on whether the B.6 had a higher or lower mortality compared to other lineages?

This is an interesting question but as we don’t have clinical data and outcomes for the cases outside our centre (and in total, we only have 62 from our centre), we don’t feel we can elaborate further about the mortality rates. On a simplistic level, the MOH reported that the mass gathering was associated with 34 deaths/3375 cases (1.0%), which is similar to the overall reported rate of 130/10219 (1.3%), but we do not have information on confounding factors such as age, underlying diseases, etc.
